# Correction: A universal method for sensitive and cell-free detection of CRISPR-associated nucleases

**DOI:** 10.1039/d0sc90196b

**Published:** 2020-09-17

**Authors:** Kurt J. Cox, Hari K. K. Subramanian, Christian Cuba Samaniego, Elisa Franco, Amit Choudhary

**Affiliations:** Chemical Biology and Therapeutics Science, Broad Institute of MIT and Harvard 415 Main Street, Rm 3012 Cambridge MA 02142 USA achoudhary@bwh.harvard.edu +1 617 715 8969 +1 617 714 7445; Department of Medicine, Harvard Medical School Boston MA 02115 USA; Divisions of Renal Medicine and Engineering, Brigham and Women’s Hospital Boston MA 02115 USA; Department of Mechanical Engineering, University of California – Riverside Riverside CA – 92521 USA efranco@engr.ucr.edu +1 951 827 2442

## Abstract

Correction for ‘A universal method for sensitive and cell-free detection of CRISPR-associated nucleases’ by Kurt J. Cox *et al.*, *Chem. Sci.*, 2019, **10**, 2653–2662, DOI: 10.1039/C8SC03426E.

In the original article, incorrect grant information from the Department of Energy was provided. The corrected Acknowledgements section is provided below, with the correct grant number:

This work was supported by the Burroughs Wellcome Fund (Career Award at the Scientific Interface to A. C.), DARPA (Brdi N66001-17-2-4055 to A. C.), NIH (1R21AI126239-01 to A. C.), Army Research Office award W911NF1610586 (to A. C.), and by the Department of Energy through grant DE-SC0010595 to E. F., which supported the salary of H. K. K. S. This work is dedicated to Professor Ronald T. Raines on the occasion of his 60th birthday.

The Royal Society of Chemistry apologises for these errors and any consequent inconvenience to authors and readers.

